# The Phytotoxicity Changes of Sewage Sludge-Amended Soils

**DOI:** 10.1007/s11270-012-1248-8

**Published:** 2012-07-04

**Authors:** Patryk Oleszczuk, Anna Malara, Izabela Jośko, Adam Lesiuk

**Affiliations:** 1 Department of Chemical Technology, University of Maria Skłodowska-Curie, pl. M. Curie-Sklodowskiej 3, 20-031 Lublin, Poland; 2 Institute of Soil Science and Environmental Management, University of Life Sciences in Lublin, Leszczyńskiego 7, 20-069 Lublin, Poland

**Keywords:** Phytotoxicity, Sewage sludge, Field experiment, Solid phase, Extracts

## Abstract

The aim of the present study was the estimation of changes in the phytotoxicity of soils amended with sewage sludge with relation to *Lepidium sativum*, *Sinapis alba* and *Sorghum saccharatum*. The study was realised in the system of a plot experiment for a period of 29 months. Samples for analyses were taken at the beginning of the experiment, and then after 5, 17 and 29 months. Two kinds of sewage sludge, with varying properties, were added to a sandy soil (soil S) or a loamy soil (soil L) at the dose of 90 t/ha. The addition of sewage sludge to the soils at the start of the experiment caused a significant reduction of both seed germination capacity and root length of the test plants, the toxic effect being distinctly related to the test plant species. With the passage of time the negative effect of sewage sludge weakened, the extent of its reduction depending both of the kind of sewage sludge applied and on the type of soil. Phytotoxicity of the soils amended with the sewage sludges was significantly lower at the end of the experiment than at the beginning. The species of the plants grown on the soils also had a significant effect on their phytotoxicity. The greatest reduction of toxicity was observed in the soil on which no plants were grown (sandy soil) and in the soil under a culture of willow (loamy soil). Solid phase of sewage sludge-amended soils was characterised by higher toxicity than their extracts.

**Electronic supplementary material:**

The online version of this article (doi:10.1007/s11270-012-1248-8) contains supplementary material, which is available to authorized users.

## Introduction

Application of sewage sludge in agriculture or for soil reclamation is an interesting solution. It is more and more frequently used in practice due to the advantageous effect of sewage sludge on the properties of soils fertilised/reclaimed with its application. Apart from soil enrichment in nutrients (Fytili and Zabaniotou 2008), an addition of sewage sludge causes an increase in organic matter content in soil (Epstein 2003). Organic matter resources in soils are relatively low and frequently require replenishment. Therefore, the use of sewage sludge in agriculture is a desirable method of their utilisation. The addition of sewage sludge to soils may thus be an inexpensive and effective alternative to the methods applied currently (mineral fertilisation, manure etc.). In spite of the undisputable advantages resulting from the application of sewage sludge in agriculture, it also involves some serious threats. Among those we should mention the presence of pathogens, heavy metals, and organic pollutants (Harrison et al. 2006; Oleszczuk 2006a; Smith 2009). That last group is particularly important, due to its diversity. That diversity is related both with the method of toxic effect, and with diversified effects on living organisms (mutagenic effect, carcinogenic effect, endocrine disrupting effect) (Singh and Agrawal 2008). With the development of analytical techniques, more and more often new potentially toxic organic compounds are identified in sewage sludge (Clarke and Smith 2011; Davis et al. 2012; Müller et al. 2006). Due to the multitude of those substances, and to the fact that their identification requires professional equipments, such contaminants are usually not the subject of routine chemical analyses. The lack of accurate information in this respect increases the risk involved in the application of sewage sludge. In this situation, biological tests may be helpful in the identification of potential threats. Biological tests permit not only measurable determination of a threat (toxic effect) but also take into account the possible interactions among the particular contaminants (antagonism/synergy effect). Moreover, the use of biological tests permits estimation of threats related with the presence of so far unidentified contaminants with potentially toxic effect. In this aspect, the estimation of phytotoxicity of sewage sludge is of particular importance due to its frequent utilisation for natural purposes. Moreover, plants are essential primary producers in the terrestrial ecosystem, whereas crop yield and quality are important success criteria in agriculture. The application of phytotoxicity tests, therefore, permits not only to evaluate the applicability of sewage sludge for agricultural or soil reclamation purposes, but also to identify potential threats for the environment and for human health.

Most of the studies concerned with the estimation of phytotoxicity of sewage sludge have been focused on the estimation of toxicity of the sewage sludge as such (Hu and Yuan 2012; Ramirez et al. 2008a; Ramirez et al. 2008b). Those studies, however, do not take into account other significant parameters, important in the assessment of the natural utilisation of sewage sludge. The toxicity of sewage sludge can also be significantly affected by the type of soil in which it is introduced (Domene et al. 2010), as well as by the kind of matrix under estimation (water extract or solid phase) (Domene et al. 2008). On the basis of studies conducted so far (Suchkova et al. 2010), it is also to be supposed that the species of plants grown can have a significant effect of the phytotoxicity of soil amended with sewage sludge, especially in the long-term approach. In spite of the importance of those problems, however, the literature lacks a comprehensive approach to those issues. Additionally, to our knowledge there have been no such investigations in the long-term approach following the introduction of sewage sludge in soil. This is of extreme importance, as it permits the estimation of risk (or the absence of risk) not only at the beginning, immediately after the introduction of sewage sludge in the soil, but also over subsequent periods. It is to be expected that with the passage of time, as a result of changes taking place within sewage sludge and interactions of pollutants with the environment, significant changes may take place in the phytotoxicity of sewage sludge.

The objective of the study was to determine the effect of the kind of plant grown, the type of soil and the kind of sewage sludge on the phytotoxicity of soils amended with sewage sludge. In the first stage of the study, estimation was made of the effect of two different soils on the phytotoxicity of sewage sludge with relation to three test plant species (*Lepidium sativum*, *Sinapis alba*, *Sorghum saccharatum*). Next, in a long-term approach, the change of phytotoxicity of soils amended with sewage sludge was estimated with relation to *L. sativum* and *S. alba* and the type of soil. Additionally, in the case of *L. sativum*, the effect of the plant species cultivated and of the kind of sewage sludge was estimated. The study involved the evaluation of toxicity of both the soil extract (US EPA) and of the solid phase of the soils, in order to achieve a more comprehensive estimation of environmental threats related with the use of sewage sludge.

## Materials and Methods

### Soils and Sewage Sludges

In the field experiment, two kinds of soils and sewage sludges were used. The soils differed in all the properties analysed (Table 1): the sandy soil (called soil S—Haplic Podzol originating from sand) was characterised by a strongly acidic pH, high content of H^+^ ions and low content of carbon and nitrogen, while the loamy soil (called soil L—Haplic Luvisol originating from silt) showed markedly better cultivation properties and was characterised by a neutral pH, good sorption qualities (CEC, TEB) and twice the nitrogen content of the soil S. The organic carbon content in the soil L was only slightly higher than in soil S (Table 1).

**Table 1:** Physico-chemical properties and heavy metals content in soils and sewage sludges used in the experiment

Properties	Soils			Sewage sludges	
		S	L	SL1	SL2
Clay	%	2	9	–	–
Silt	%	8	75	–	–
Sand	%	90	16	–	–
pH	in KCl	3.6	7.1	6.1	7.6
CEC	mmol∙kg^−1^	20	190	440	976
TOC	g∙kg^−1^	8.3	10.7	188.2	157.2
N_t_	g∙kg^−1^	0.8	1.5	40.6	22.1
Cd	mg∙kg^−1^	0.17	0.37	0.97	1.05
Zn	mg∙kg^−1^	18.8	39.6	898	808
Pb	mg∙kg^−1^	27.6	34.1	46.8	37.4
Cr	mg∙kg^−1^	5.97	17.2	19.9	22.2
Cu	mg∙kg^−1^	–	2.06	96	137
Ni	mg∙kg^−1^	–	5.66	14.3	14.3

SL1 and SL2 refer to sewage sludges

*S* sandy soil, *L* loamy soil, *pH* reactivity, *CEC* cation exchange capacity, *TEB* the total of the exchangeable bases, *TOC* total organic carbon content, *N*_*t*_ total nitrogen content

Two different sewage treatment plants (SL1 and SL2) localized in southeast part of Poland (Zamość—SL1 and Biłgoraj—SL2) were selected to collect sewage sludges. The selected treatment plants were characterised by their catchment area (indicated by the number of inhabitants and amount of sewage treated) as well as by the industrial character of the area (quantity and variety of industrial plants). Sewage treatment plants treat about 12,500 (SL1) and 4,500 (SL2) m^3^ day^−1^, respectively, of municipal wastewater. The sewage sludges were collected at the end point of the sewage sludge digestion process. Sewage sludges were typical aerobically digested. As in the case of the soils, the sludges differed in most of the parameters studied. Sludge SL1 was characterised by a lower CEC and a lower pH compared to sludge SL2. In sludge SL2, a higher content of organic matter and nitrogen was noted, by 16.5 % and 45.6 %, respectively, relative to sludge SL1.

### Field Experiment and Soil Sampling and Preparation

The present experiment was carried out in two blocks of experimental plots (15 m^2^) divided according to the type of soil used (sandy and loamy soil). Additionally, each of the blocks was divided into two sub-blocks into which the two kinds of sewage sludge (SL1 and SL2) were introduced at the dose of 90 t/ha. Sludge doses were calculated taking into consideration the sludge’s dry mass and the density of the solid soil phase. Sewage sludges were mixed with a surface soil layer up to a depth of 20 cm. Grass mixture (e.g. *Lolium perenne*, *Festuca pratensis*, *Lolium multiflorum* and *Phleum pratense*) was cultivated on soils. In addition, in the experiment with sewage sludge SL1, in parallel to the grass mixture also other plants were cultivated: clover (*Trifolium pratense*), willow (*Salix viminalis*) as well as a control without plants. These plants were chosen for several reasons. Willows are used for short-rotation coppices because they grow rapidly (2–3 m per year), are easy to propagate, and yield high biomass when planted at high densities. Willows can be used for different purposes, for example as carbon dioxide neutral biofuel. Perennial ryegrass is an important pasture and forage plant, and is used in many pasture seed mixes. In fertile soil, it produces a high grass yield, which is frequently sown for short-term ley grassland, often with red or white clover (*T. pratense*).

The experiment was carried out for 29 months. Soil samples were collected for analysis after the introduction of sewage sludge, and then after 7, 19 and 29 months. Control soil (non-amended) and sewage sludge-amended soil samples were collected from the level of 0–20 cm with a (5 cm i.d., 60 cm) stainless steel corer. Six independent samples (replicates) were taken from each plot. The samples were transported to the laboratory, air-dried in air-conditioned storage rooms (about 25 °C) for several days (in darkness), manually crushed and sieved (<2 mm) prior to chemical analyses.

### Phytotoxicity Test

Sewage sludge-amended soils toxicity was assessed with the commercial toxicity bioassay—Phytotoxkit^™^ Test (Phytotoxkit 2004). The phytotoxkit microbiotest measures the decrease (or the absence) of seed germination and of the growth of the young roots after 3 days of exposure of seeds of selected higher plants to contaminated matrix in comparison to the controls in a reference soil. Ten seeds of each plant were positioned at equal distance near the middle ridge of the test plate on a filter paper placed on top of the hydrated soil. After closing, the test plates were placed vertically in a holder and incubated at 25 °C by 3 days. At the end of the incubation period, a digital picture was taken of the test plates with the germinated plants. The analyses and the length measurements were performed using the Image Tool 3.0 for Windows (UTHSCSA, San Antonio, USA). The bioassays were performed in three replicates. The percent inhibition of seed germination (SG) and root growth inhibition (RI) were calculated with the formula:

$$ SG/RI = \left( {\frac{{A - B}}{A}} \right) \times 100 $$

where, A—mean seed germinaton and root length in the control soil/soil-sewage sludge mixture; B—mean seed germination and root length in the tested soil/soil-sewage sludge mixture.

To evaluate the effect of extracts of sewage sludge-amended soils on plants, the test was based on a germination/elongation recommended by OECD (1984). Water extracts were obtained according to the EN 12457-2 protocol (EC 2002). The experimental conditions of test are described in Table S1 (supporting information).

### Chemical Analysis

The chemical properties of sewage sludges and soils studied were determined by standard methods. The pH was measured potentiometrically in 1 M KCl after 24 h in the liquid/soil ratio of 10; the cation exchange capacity (CEC) was determined in the 0.1 N HCl extract. The total nitrogen (N_t_) was determined by the Kjeldahl’s method (van Reeuwijk 1995) without the application of Dewarda’s alloy (Cu–Al–Zn alloy-reducer of nitrites and nitrates). Total organic carbon was determined by TOC-V_CSH_ (SHIMADZU) with Solid Sample Module SSM-5000.

## Results

### Phytotoxicity of Soils and Sewage Sludges to Different Plants

**Fig. 1:**
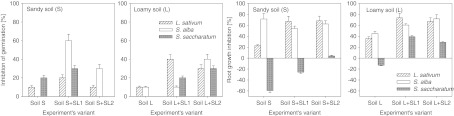
Inhibition of germination and root growth of *L. sativum*, *S. saccharatum* and *S. alba* in control sandy (Soil S) and loamy (Soil L) soil and sewage sludges (SL1 and SL2)-amended soils. Sewage sludges dose—3 %

Figure 1 presents the effect of the soils studied and of the sewage sludge-amended soils on the inhibition of seed germination and root growth as related to the test plants. The effect of the soils studied on seed germination was varied and depended on the plant species (Fig. 1). In the control soil S, the highest sensitivity was observed for *S. saccharatum*, for which seed germination was inhibited at the level of 20 %. Values lower by half were observed for *L. sativum*; while in the case of *S. alba*, no negative effect of soil S on the germination of that plant was observed. Totally different results were obtained for soil L. The highest sensitivity (10 % inhibition of germination) was noted in the case of *L. sativum* and *S. alba*. Germination of *S. saccharatum* in soil L was at the level of 100 % (Fig. 1).

The results obtained in the case of root growth inhibition did not support those related to germination inhibition (Fig. 1). In soil S, the highest sensitivity was observed for *S. alba* (70 % root growth inhibition). Several-fold lower values were obtained for *L. sativum*; while in the case of *S. saccharatum*, a positive effect of the soil S on root growth was observed. In the control soil L also a negative effect of the soil was observed in the case of root growth of *L. sativum* and *S. alba* (by 35.9 % and 45.0 %, respectively). The effect of soil L on the growth of roots of *S. saccharatum*, as in the case of soil S, was positive though not as significant as that observed in soil S.

In most cases the addition of sewage sludges to the soils had a significant (*P* ≤ .0.05) negative effect on both seed germination and root growth with relation to all plants studied (Fig. 1). The most distinct inhibiting effect of sewage sludge was observed for *S. alba* and soil S. The addition of sludge SL1 as well as SL2 caused significant inhibition of germination of the plant at levels of 60 % and 30 %, respectively. A negative effect of sewage sludge addition was noted also for *L. sativum* and *S. saccharatum*, but only in relation to sludge SL1. The values determined for sludge SL2 did not differ significantly (*L. sativum*) or were lower (*S. saccharatum*) than those noted for the control soil S (not amended with the sludge). In soil L a distinct negative effect of sewage sludge was observed with relation to all of the tested plants. A greater negative influence (with the exception of *L. sativum*) was observed after the application of sludge SL2 compared to SL1.

The application of sewage sludge SL1 as well as SL2 to soil S caused similar inhibition of the growth of roots of *L. sativum*, at the level of 60 % (Fig. 1). Also, both kinds of sewage sludge had a reducing effect on the positive effect of soil S on root growth of *S. saccharatum*. However, no significant differences were found for *S. alba* root growth inhibition between soil S with sewage sludge addition and the same soil without such an amendment (Fig. 1). The application of the sewage sludge kinds under study in soil L caused a significant reduction of the length of roots of the plants tested. The highest sensitivity in this respect was characteristic of *L. sativum*. The root growth inhibition after the application of sewage sludge was greater by 105 % (SL1) and 86 % (SL2) than that observed for soil L with no sewage sludge addition (Fig. 1).

### Changes of the Sewage Sludge-Amended Soil Phytotoxicity to Different Plants

**Fig. 2:**
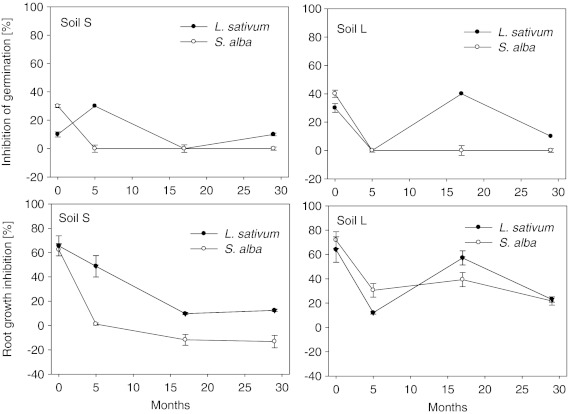
Changes of the germination and root growth inhibition of *L. sativum* and *S. alba* in sewage sludge-amended soils. Sewage sludge SL2 was applied to the soil at the dose of 3 %

Figure 2 presents the change in phytotoxicity of soils amended with sewage sludge with relation to the plant tested and to the time of analyses. Due to their relatively high sensitivity, *L. sativum* and *S. alba* were selected for further tests. Both in the case of germination and of root growth inhibition significant differences were observed between the species tested. A greater dynamics of changes in relation to the terms was characteristic of *L. sativum* as compared to *S. alba* (Fig. 2). Moreover, for most of the terms *L. sativum* displayed higher sensitivity to sewage sludge than *S. alba*, which does not support the observations from the beginning of the experiment, when greater values of inhibition of seed germination were noted for *S. alba* than for *L. sativum*.

In the case of *S. alba*, in both soils a significant decrease in the inhibition of seed germination was observed at the beginning of the experiment, to a level indicating a lack of the toxic effect (Fig. 2). Until the end of the experiment that value remained at a constant level without any significant changes.

In the case of *L. sativum*, seed germination depended on the soil type. In soil S, an increase of toxicity was noted (after 6 months), followed by a significant reduction of the inhibition of seed germination to a level indicating an absence of the toxic effect. On the final date of the experiment, there was another increase to the level from the beginning of the experiment (10 % inhibition of seed germination). In soil L, a reverse tendency was observed. After the initial months a decrease of toxicity was noted, then a significant increase, followed by another drop of toxicity. The value recorded on the final date of the experiment (10 %) was significantly lower than at the beginning of the experiment (30 %).

As it was observed in the case of inhibition of seed germination, also root growth inhibition was determined by the soil type. In soil S with an addition of sewage sludge, both in the case of *L. sativum* and *S. alba* a gradual decrease of root growth inhibition in time was observed (Fig. 2). *L. sativum* displayed greater sensitivity to the presence of the sludge than *S. alba* on all the dates of analyses (with the exception of the beginning of the experiment). Totally different results were recorded in soil L. As in soil S, both of the tested plants displayed the same tendency, but both the range and the direction of changes were completely different than in soil S. After an initial significant decrease of root growth inhibition, a significant increase was observed, followed by another decrease both in the case of *L. sativum* and *S. alba*. Those changes were clearly correlated with the inhibition of seed germination that was observed for *L. sativum* in soil L.

### Changes of Phytotoxicity Depending on Soil and Sewage Sludge Type

**Fig. 3:**
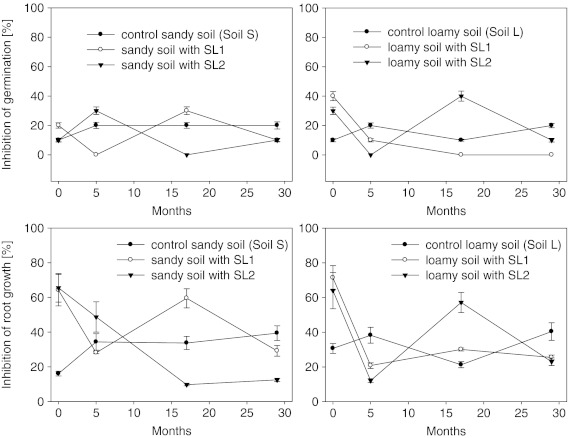
Changes of *L. sativum* inhibition of germination (*top figures*) and the root growth inhibition (*bottom figures*) in sewage sludge-amended soil depending on the type of soil (sandy and loamy) and sewage sludge (SL1 and SL2). Sewage sludges were applied at the dose 3 %

As demonstrated before, the level of changes in both seed germination and root length varied in relation to the soil type and to the test plant species (Fig. 2). Figure 3 presents the changes of phytotoxicity to *L. sativum* with relation to the kind of sewage sludge applied in the soils under study. In the control soil S a significant increase of toxicity (to the level of 20 %) was observed only during the initial 6 months from the beginning of the experiment. On later dates of analyses the phytotoxicity in that soil remained at a constant level. In the control soil L the inhibition of germination of *L. sativum* was subject to continuing oscillations within the range from 10 % to 20 % (Fig. 3) throughout the duration of the experiment.

The effect of sewage sludge added to the studied soils on seed germination of *L. sativum* was varied both with relation to the kind of sludge and to the soil type. The toxicity of soil S after the application of the sewage sludges was different almost on every date of the experiment (Fig. 3). However, ultimately, on the final date of the experiment, no difference was noted in the seed germination inhibition values between the two kinds of sewage sludge. Also, the results obtained indicated lower toxicity of the sludge-amended soils compared to the control soil S. A totally different trend was observed in the case of soil L fertilised with sludge SL1 and sludge SL2. After an initial decrease of the inhibition of seed germination (Fig. 3), the decreasing tendency continued only in the soil amended with sludge SL1. In soil L fertilised with sludge SL2, after 18 months of the experiment, an increase of the inhibition of seed germination was observed, to a level significantly exceeding the values recorded for the control soil. As in soil S, on the final date of the experiment, the inhibition of seed germination was lower in soil L amended with sewage sludge than in the control soil.

The addition of sewage sludge had also a significant effect on root growth inhibition (Fig. 3). The addition of sludge SL1 to soil S was characterised by the same trend that was observed in the case of inhibition of seed germination. The range of differences between the dates of analyses in the case of inhibition of root growth was, however, notably greater than that observed for the inhibition of seed germination. In soil S with an addition of sludge SL2, a decrease was observed that lasted from the beginning of the experiment till month 17th. Between the 17th month and the final date of the experiment, no significant differences were noted (Fig. 3). In soil L, after the addition of sludge SL1 as well as of SL2, the root growth inhibition displayed the same tendency (Fig. 3). Whereas, there were differences between the two kinds of sludge, resulting from the magnitude of the effect exerted by them after 17 months of the experiment. In the case of the remaining dates of analyses, the values of root growth inhibition caused by sludge SL1 and SL2 were highly similar (Fig. 3).

As it was observed in the case of inhibition of seed germination, on the final date of experiment root growth inhibition was lower in soils S and L with an addition of sewage sludge than in the control soils. Reduction of root growth inhibition in relation to the beginning of the experiment was fairly constant in soil L, irrespective of the kind of the sewage sludge applied, attaining the level of 64 %. In soil S, on the other hand, significant differences were observed in relation to the kind of sludge applied. Better reduction of root growth inhibition after 30 months of the experiment was observed in the soil amended with sludge SL2 (by 81 %) than in that fertilised with sludge SL1 (by 55 %).

### Effect of Plants on Phytotoxicity Changes in Sewage Sludge-Amended Soil

**Fig. 4:**
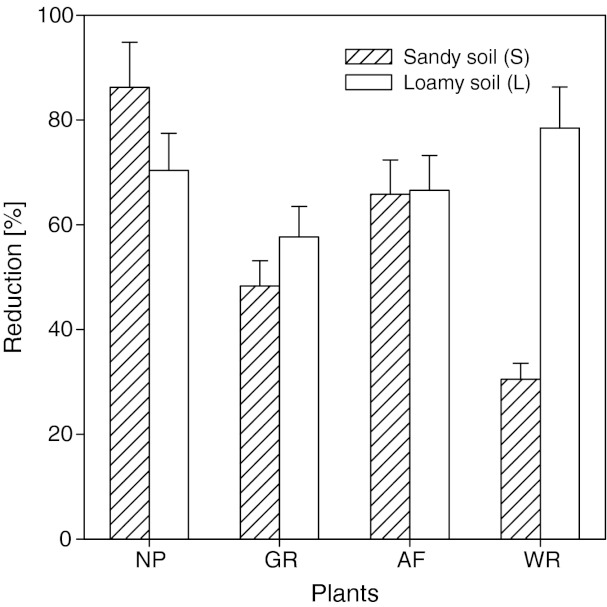
The reduction [%] of root growth inhibition in sewage sludge-amended soils after 29 months comparing to beginning of the experiment. Sewage sludge SL1 was applied to the soils at the dose 3 %. *NP* no plants, *GR* mix of the grasses, *AF* alfalfa, *WR* wicker

All of the plants cultivated in the experiment significantly determined the phytotoxicity of soil amended with sewage sludge. That effect varied in relation to the soil type. Only in the case of the mix of grasses the values of root growth inhibition in both of the soils under study were highly similar (Figure S1, supporting information). In the other experimental treatments, the particular changes were specific for a given variant of the experiment. Depending on the soil type, the reduction of the toxic effect varied from 31 % to 86 % in soil S, and from 58 % to 79 % in soil L (Fig. 4). The greatest reduction of the toxic effect was observed in the treatment with no plants in soil S (86 %), while in soil L it was in the treatment with wicker (79 %). Whereas, willow had a relatively unfavourable effect on the reduction of the toxic effect in soil S, attaining values that were the lowest among all the experimental treatments (31 %).

### Phytotoxicity of Extracts from Sewage Sludge-Amended Soil Comparing to Solid Phase Toxicity

**Fig. 5:**
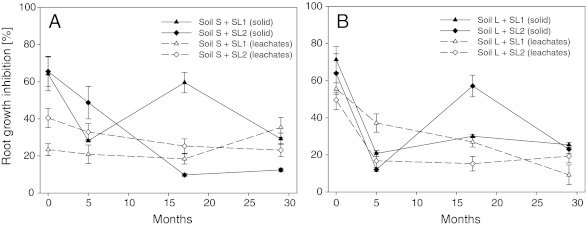
Comparing of the extracts toxicity of sewage sludge-amended soils with solid phase toxicity to *L. sativum*. **a** Sandy soil (S). **b** Loamy soil (L)

Figure 5 presents changes in root growth inhibition of water extract obtained from soils amended with sewage sludge against the background of root growth inhibition of solid phase. The difference in toxicity between solid phase and extracts was clearly dependent on the terms of experiment. In all experimental treatments, on the first date of the experiment, higher inhibition of root growth was observed for the solid phase rather than the extracts. On the second date of the experiment, that tendency persisted only in soil S; while in soil L, a reverse direction of changes was noted, which indicated higher toxicity of the extracts compared to the solid phase. On later dates of the experiment, those relations were largely determined by the soil type and the kind of sewage sludge introduced into soils. In soil S, on the third date of the experiment, extracts displayed greater toxicity in the case of sludge SL2, while a reverse tendency was noted for sludge SL1. Differences between the two kinds of sewage sludge were observed also in soil L. In this case, however, solid phase displayed greater toxicity than extracts from soil amended with sludge SL2. In the case of sludge SL1, no significant differences were found between the toxicity of extracts and solid phase. The difference in toxicity between extracts and solid phase on the final date of the experiment was also determined by the type of soil. In soil S, irrespective of the kind of sewage sludge, higher values of root growth inhibition were noted in the case of extracts than for the solid phase; while in soil L, solid phase was more toxic than the extracts. Noteworthy is the fact that the differences in toxicity between extracts and solid phase were significantly less pronounced than those observed at the beginning of the experiment.

## Discussion

The problem of the effect of sewage sludge on seed germination and plant growth has been addressed by numerous researchers (Fjällborg and Dave 2004; Fuentes et al. 2006; Hu and Yuan 2012; Oleszczuk 2008; Ramirez et al. 2008a). However, those studies were focused primarily on the estimation of the toxicity of sewage sludge as such, without taking into account other important parameters that may be of major significance in the utilisation of such material. Soil type, plant species used in the biotests, kind of sewage sludge, effect of plants grown on soil amended with sewage sludge (especially if a given study addresses the problem in a long-term approach) or the kind of matrix tested (extracts, solid phase) are highly important issues whose role should be studied. The study presented here shows that in most cases the addition of sewage sludge to soils caused an increase in their phytotoxicity comparing to non-amended soil (Fig. 1). A distinct increase of the toxic effect after the application of sewage sludge was observed for all three test plants: *L. sativum*, *S. alba* and *S. saccharatum*. The negative effect of sludge was more pronounced in relation to root growth inhibition than to seed germination. This supports earlier research (Fuentes et al. 2006) in which root growth inhibition was a more sensitive indicator of toxicity than seed germination. It was observed, however, that soil type had a stronger effect on the differences between the particular test plants in the case of seed germination than of root growth inhibition (Fig. 1).

Although at the beginning of the experiment the differences between the plant species tested were slight, with the passage of time they were amplified. While in the case of *S. alba*, the decrease in toxicity of soils amended with sewage sludge was gradual (initially a significant decrease and then either a slow decline or a lack of significant change); in the case of *L. sativum*, significant variations were observed almost throughout the period of the experiment (Fig. 2). It should also be emphasised that a stronger negative effect exerted by soils amended with sewage sludge of the particular dates of experiment was observed more often in the case of *L. sativum* than of *S. alba*. In the case of *L. sativum*, the changes observed were determined to a greater extent by the soil type than was for *S. alba*, for which the soil type was of notably lesser importance. This may indicate greater sensitivity of *L. sativum* than *S. alba* to the pollutants occurred in sewage sludge and to changing environmental conditions. Contaminants present in sewage sludge, due to the mineralisation of organic matter, are subject to continuous processes of remobilisation and repeated binding by newly formed organic structures, which affects their bioavailability and, at the same time, their toxicity (Oleszczuk 2006b). Research indicates that *L. sativum* is one of those plants which are exceptionally sensitive to the presence of many contaminants, organic as well as inorganic (Heemsbergen et al. 2009). Moreover, it has been demonstrated that the plant can be particularly useful in the estimation of toxicity of sewage sludge (Oleszczuk 2008, 2010). *L. sativum* sensitivity to the presence of toxic substances has been confirmed in numerous articles (Fritz 1999; Fuentes et al. 2006; Walter et al. 2006). In a recent study (Oleszczuk 2010), in which the effect of various kinds of sewage sludge on their toxicity was tested in relation to ten different plant species, *L. sativum* was also characterised by the highest sensitivity.

Analysis of changes in phytotoxicity revealed its notable dynamics, irrespective of the plant tested and the kind of sewage sludge applied (Fig. 2). Additionally, the application of the same sewage sludge in different soils showed that the direction of changes of toxicity was related with the type of soil (Fig. 3). This provides an extremely important piece of information, namely, that the same kind of sewage sludge can display various levels of toxicity depending on the soil type. It should also be emphasised that the direction of changes of phytotoxicity may differ in relation to soil type. While at the beginning of the experiment the differences between the individual plant species (especially in the case of root growth inhibition) were very small, with the passage of time they were notably amplified. A study on the effect of soil type on the toxicity of sewage sludge with relation to collembolans was conducted earlier by Domene et al. (2010). Those authors found a significant relationship between soil properties and the toxic effect towards the tested organisms. The results obtained in this study indicate that also with relation to plants the type of soil has a significance in the estimation of the toxicity of sewage sludge, but its effect is more diversified in the case of seed germination than of root growth inhibition. Moreover, the type of soil determined its toxicity with relation to sewage sludge to a greater extent as a long-term effect than at the very beginning of experiments. On the basis of the results obtained, however, it was not possible to conclude definitively which of the soils studied displayed greater toxicity after amendment with sewage sludge. It is to be supposed that the observed toxicity was specific for the given conditions and that, depending on the soil and sewage sludge, their different properties may have had a multidirectional influence on the overall toxicity of a sewage sludge-amended soil.

The changes in the phytotoxicity of the studied soils, observed over time, were most likely related with the transformations, mentioned earlier in the text, to which the pollutants present in the sewage sludge applied to the soils were subject. A number of studies concerning the fate of organic and inorganic pollutants in soils amended with sewage sludge indicate that those compounds undergo a variety of processes (e.g. adsorption, desorption, bioformation, volatilisation, photodegradation, bioaccumulation, leaching and incorporation into humic substances structures (sequestration or bound residue formation); De Jonge et al. 2002; Hesselsøe et al. 2001; La Guardia et al. 2001). Those processes significantly determine the bioavailability of the pollutants (Alexander 2000) and, indirectly, also their toxicity. After 29 months, in almost all treatments a significant decrease of phytotoxicity was observed in soils amended with sewage sludge in relation to the beginning of the experiment. Most probably that was a result of combination of all of the processes mentioned above. Moreover decreasing of the toxicity of the sewage sludge-amended soil could be related with quickly degraded labile compounds which very often occurs in sewage sludges and act as phytotoxins. The increase of phytotoxicity observed after 5 months in most of the experimental treatments (Fig. 3) was most probably related with remobilisation of pollutants, fairly frequently observed in soils amended with sewage sludge or composts (Oleszczuk 2006b). Terry et al. (1979) and Rowell et al. (2001) stated that 26–42 % of the organic matter introduced together with the sludge underwent mineralisation very quickly. As a result of that process, formerly unavailable pollutants related with organic matter undergo remobilisation. An increase in the phytotoxicity after 5 months was most probably related to the fact that the organic contaminants, initially adsorbed to the sewage sludges/soil mixture, were temporarily less available. As a result of organic matter mineralization, the strength of these bonds could weaken, and hence, there was an increase in the bioavailability of pollutants which had not been bioavailable earlier (Oleszczuk 2006b).

Apart from the determination of the effect of time on the change (increase or lowering) of the toxicity of soils amended with sewage sludge, the effect of the kind of plants cultivated on sludge-amended soils is another important issue. In spite of the significance of these problems, especially from the practical point of view, no studies of this type have been undertaken so far. It is known that the rhizosphere can play an important role in the degradation of many organic contaminants (Megharaj et al. 2011). Moreover, plants can accumulate contaminants, which are frequently used in the techniques of phytoremediation (Wu et al. 2010). Those processes cause a lowering of the concentration of pollutants, and thus also of the toxicity of soils amended with sewage sludge. Significant differences between the soils were observed only in the case of wicker. For the remaining test plants, the differences between the soils were not statistically significant. The values obtained, however, differed among the particular test plants, which indicate their varied effect on the soils under study. Nevertheless, on the basis of the results obtained it is difficult to propose an explanation of the observed phenomena. Still, the study has an important practical aspect, as it shows that through suitable choice of land management system it is possible to achieve a reduction of the phytotoxicity of soils amended with sewage sludge.

It is commonly accepted that toxicity largely depends on the water solubility of pollutants. Biological tests are a useful tool for the control of wastes that are to be applied in soil, but another important question is whether the tests should be performed for the whole matrix of for the soil extract (Ramirez et al. 2008a). However, studies on the sewage sludge phytotoxicity concentrate mainly on the analysis of water extracts (Fuentes et al. 2006; Mantis et al. 2005; Wong et al. 2001). The application of water extracts provides important information; however, they do not give a fully comprehensive description of the toxicity of sewage sludges. The results obtained in this study showed clearly that the analysis of extracts is not sufficient for the full characterisation of risks related with sewage sludge. This is also supported by earlier studies conducted with relation of other organisms and environmental matrices (Chial and Persoone 2003; Ramirez et al. 2008a). In this study, the values obtained for the solid phase were several-fold greater than those for the extracts (Figs. 1–5). In this respect, the type of soil in which the sewage sludge was applied had a significant effect. Only on the final date of the experiment the differences between the solid phase and the extracts were similar, which most probably resulted from reduced concentration of pollutants with low water-solubility, the remaining toxicity being related with the soluble fraction of the pollutants.

## Conclusions

Sewage sludge to be utilised in agriculture must be subjected to comprehensive evaluation comprising not only the determination of the basic physicochemical properties, content of pollutants or pathogenic bacteria, but also of the ecotoxicological properties. As a result of the study presented here, extremely important information was acquired that, in certain cases, supported earlier observations and provided data of vast importance from the viewpoint of the use of sewage sludge as a fertiliser: (1) phytotoxicity of sewage sludge and its changes over time are significantly determined by the soil type. In this case, the soil type is one of the most important factors regulating the phytotoxicity of sewage sludge, especially in the long-term aspect; (2) with the passage of time the phytotoxicity of soils amended with sewage sludge undergoes a change, not always in a direction causing a lowering of their toxicity. The extent of particular changes depends both on the properties of the soils and on the kind of sewage sludge, and for various kinds of sludge, it can have different direction in a single soil; (3) the extent of changes in the toxic effect on the test plants is related to the species of the plant under cultivation; (4) in spite of similar initial toxicity displayed by various kinds of sewage sludge in relation to particular plants, further changes of phytotoxicity may vary with relation to the sewage sludge and the type of soil; (5) the intensity of toxicity towards various plants depends not only on the kind of sewage sludge but also on the soil type; (5) proper selection of the conditions of management sludge-amended soil can be conducive to a reduction of phytotoxicity; and (6) estimation of extracts is not sufficient for the full characterisation of the risk involved in the utilisation of sewage sludge in agriculture.

## Electronic Supplementary Material

Below is the link to the electronic supplementary material.

ESM 1 (PDF 61 kb)
