# Supplementary material for: The Phytotoxicity Changes of Sewage Sludge-Amended Soils
Source: Water Air Soil Pollut. 2012 Jul 4;223(8):4937–48. doi: 10.1007/s11270-012-1248-8 (PMC3443479; doi:10.1007/s11270-012-1248-8)
Supplement: Supplementary file 1 — (PDF 61 kb) [file 11270_2012_1248_MOESM1_ESM.pdf]

supporting information
Click here to download attachment to manuscript: Supporting information.doc Click here to view linked References

Supporting information:

THE PHYTOTOXICITY CHANGES OF SEWAGE SLUDGE-AMENDED SOILS

Patryk Oleszczuk[1*], Izabela Jośko[1], Anna Malara[1], Adam Lesiuk[2]

[1]*Institute of Soil Science and Environmental Management, University of Life Sciences in Lublin, Leszczyńskiego 7, 20-069 Lublin, Poland*

[2]*Department of Chemical Technology, University of Maria Skłodowska-Curie, pl. M. Curie-Sklodowskiej 3, 20-031 Lublin, Poland*

* *patryk.oleszczuk@up.lublin.pl*

Journal: Water Air and Soil Pollution
Number of pages: 4
Number of tables: 1
Number of figures: 1

Table S1. Description of the leaching procedure (EC, 2002)

Origin	EN 12457-2
Method	Batch extraction
Particle size (mm)	<4
Leaching medium	DIWater
Liquid-to-solid ration (L/S)	10
Number of steps	1
Method of agitation	Rotation
Rotation speed (rpm)	10
Contact time (h)	24
Filter pore size (μm)	0.45

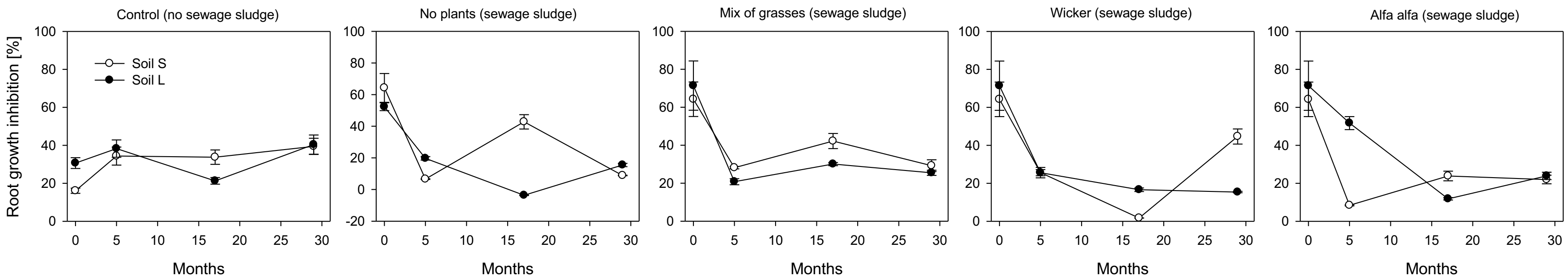

Figure S1. Changes of root growth inhibition in sewage sludge-amended soils depending on plants cultivated.

EC 2002 Council Decision of 19 December 2002 establishing criteria and procedures for the acceptance of waste at landfills pursuant to Article 16 of and Annex II to Directive 1999/31/EC. 2003/33/EC, OJ L11, 27.
